# The correlation between expression profiles measured in single cells and in traditional bulk samples

**DOI:** 10.1038/srep37022

**Published:** 2016-11-16

**Authors:** David Dzamba, Lukas Valihrach, Mikael Kubista, Miroslava Anderova

**Affiliations:** 1 Department of Cellular Neurophysiology, Institute of Experimental Medicine, Academy of Sciences of the Czech Republic, Prague, Czech Republic; 2 2^nd^ Faculty of Medicine, Charles University, Prague, Czech Republic; 3 Laboratory of Gene Expression, Institute of Biotechnology, Academy of Sciences of the Czech Republic, BIOCEV, Vestec, Czech Republic

## Abstract

Reverse transcription quantitative PCR (RT-qPCR) is already an established tool for mRNA detection and quantification. Since recently, this technique has been successfully employed for gene expression analyses, and also in individual cells (single cell RT-qPCR). Although the advantages of single cell measurements have been proven several times, a study correlating the expression measured on single cells, and in bulk samples consisting of a large number of cells, has been missing. Here, we collected a large data set to explore the relation between gene expression measured in single cells and in bulk samples, reflected by qPCR Cq values. We measured the expression of 95 genes in 12 bulk samples, each containing thousands of astrocytes, and also in 693 individual astrocytes. Combining the data, we described the relation between Cq values measured in bulk samples with either the percentage of the single cells that express the given genes, or the average expression of the genes across the single cells. We show that data obtained with single cell RT-qPCR are fully consistent with measurements in bulk samples. Our results further provide a base for quality control in single cell expression profiling, and bring new insights into the biological process of cellular expression.

Single cell analysis methods have been recently shown to gain an increasing importance when biological complexity is studied^1^. The unique position of these techniques was highlighted when the single cell RNA/DNA sequencing was awarded as the method of the year 2013 in Nature Methods^2^. Although, there is no doubt that single cell sequencing is revolutionizing our capacity to understand and describe biological diversity^3^, there are still some limits preventing its wide exploitation^4^. In comparison to that, the reverse transcription quantitative polymerase chain reaction, RT-qPCR, is a routine and cost-effective method for precise and accurate mRNA analysis^5^,^6^. For its broad application and high level of standardization, RT-qPCR is described as a “gold” standard method for mRNA quantification, often used to validate the results achieved by other techniques^7^. The advantages of RT-qPCR are its high sensitivity, specificity and excellent reproducibility.

Traditionally, gene expression has been studied in samples that may be composed of many different cell types. The measured expression is then a combined response of all the cells which may mask the possible response of a particular cell type. Specific fluorescent labeling allows cells of a certain type to be purified and analyzed. If analyzed in bulk, the measured results will still be the average of all the cells present^8^ and variations due to fluctuations, local environment, stimuli and other effects may go unnoticed. To study these variations, single cell expression profiling was introduced^9^. Single cell RT-qPCR allows the identification and characterization of rare cells, exploration of population heterogeneity and the finding of correlations between gene expressions on the cellular level^10^,^11^,^12^. Expression profiling of single cells revealed that gene expression occurs in burst^13^,^14^ resulting in the existence of small population of cells having high expression and large population expressing very small number of transcripts. When the histogram showing the distribution of transcripts of a certain gene is transformed into the log-scale, it can be fitted by Gaussian curve describing the normal distribution^9^.

Expression profiling in single cells by RT-qPCR are challenging measurements^15^, and concerns about the conclusions made have occasionally been raised. To address some of these concerns, we have performed an extensive comparison of gene expression measured in single astrocytes during ageing, with the gene expression measured in classical bulk samples containing thousands of astrocytes. We show that the single cell data, when collected and pre-processed appropriately, are fully consistent with measurements in bulk samples. This means that the manipulation of single cells, which involves collection of cells by fluorescence-activated cell sorter (FACS), the RT-qPCR preamplification workflow, data analysis, data pretreatment and handling of missing data, have a negligible influence on the gene expression in the individual cells. Based on this comparison, we design a quality control assessment and validation scheme of the measured data, which provides a new valuable dimension to the interpretation of single cell expression data.

## Results and Discussion

In this study we measured the expression of 95 genes in cortical astrocytes isolated from 1-, 3–4-, 10- and 20–23-month-old GFAP/EGFP mice (three at each time point: 1 M.1-3, 3–4 M.1-3, 10 M.1-3 and 20–23 M.1-3), in which the expression of enhanced green fluorescent protein (EGFP) is controlled by the human promoter for glial fibrillary acidic protein (GFAP)^16^. From each mouse, single astrocytes and bulk astrocyte samples were collected and their expression profiles were measured. In total, the final data set contained over 66 000 data points (12 bulk samples + 693 single cells multiplied by 95 genes). For the list of measured genes and assay primer sequences, see Supplementary Table S1.

### The relation between single cell and bulk gene expression data

**Figure 1:**
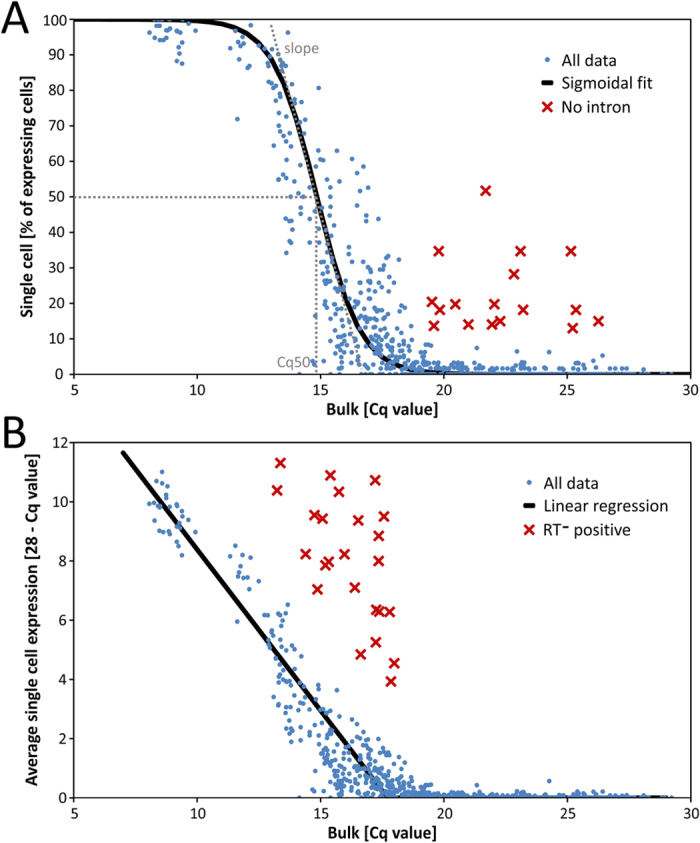
Correlation between gene expression measured in single cells and in bulk samples. Data describing the expression in single cells and in bulk samples were plotted in two-dimensional graphs and fitted by sigmoidal and linear functions. The functions show that single cell data are fully consistent with measurements in bulk samples and also provide a tool for quality control assessment. (**A**) Sigmoidal function describing the correlation between the fraction of cells expressing a gene and the bulk Cq values. Blue data points represent data obtained with intron spanning primers suppressing the signal from genomic background. Red crosses represent data generated by primers that do not span introns and may amplify gDNA (these were not included in the curve fitting). Basic parameters of the sigmoidal function are highlighted in the figure. (**B**) Linear function describing the correlation between the average Cq of single cells and bulk Cq values. The average Cq value of single cells is expressed as the maximal specific signal on the Biomark platform (Cq 28) minus the average Cq calculated from all single cells where the missing values were replaced by the largest Cq measured for the particular gene + 2. Blue data points represent data used for the linear fit. Red crosses represent data giving positive signal in RT minus controls.

The aim of this study was to describe the relation between gene expression measured in single cells and in bulk samples, using RT-qPCR. We modelled this relation using two different approaches. In both we considered only the data obtained with intron spanning primers, which suppresses background due to the genomic DNA present. In the first approach, for each gene we compared the expression in bulk samples expressed in Cq values, with the fraction of single cells that have detectable expression of these genes. This relation is shown in Fig. 1A, where each data point represents one out of the 70 genes measured in one out of the twelve mice (70 × 12 = 840 in total). The data were fitted with the sigmoidal function:


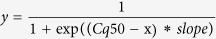


where *x* represents the bulk Cq value, *y* represents the fraction of genes expressing single cells, *Cq50* is the bulk Cq value that corresponds to gene expression in 50% of the cells, and the *slope* is the derivative of the sigmoidal curve at *Cq50*. For the astrocytes in our experiment *Cq50* was 14.85 cycles and the *slope* was −1.12 percent per cycle.

In the second approach we compared the bulk Cq values with the average of the single cell gene expression, expressed as 28 – Cq (as 28 cycles is considered the detection limit corresponding to a single template molecule, or less for samples analyzed on the Biomark platform; negative values were replaced with 0). To reduce bias introduced when calculating average Cq values for the transcripts in the single cells due to missing data, these were replaced by the largest Cq measured for the particular gene in a single cell + 2^15^. This relation is expected to be linear and indeed it is for the high and medium expressed genes (Fig. 1B). Transcripts from very low expressed genes were not detected in any of the single cells, even though they were found to be expressed in bulk samples. This is due to the relatively small number of single cells characterized in each measurement (tens of single cells) compared to thousands of cells in the bulk samples. Consequently, low level transcripts may then go undetected in single cells, because transcripts are not present in any of the cells analyzed. Noteworthy, the expression profiles of single cells and bulk samples in Fig. 1A,B were obtained using different RNA extraction protocols (the direct lysis for single cells vs. column-based extraction protocol for bulk samples). However, as was shown these protocols differ only in extraction efficiency, keeping the expression profiles correlated^17^. Therefore the only effect of different extraction protocols is a scale factor, which could offset the data but does not affect conclusions.

The observed relations show that single cell profiling data are fully consistent with those measured in bulk samples. Therefore, the additional information obtained from the single cell profiling about the heterogeneity among the cells should be biologically relevant. The only limiting aspect of single cell profiling is that very low expression, that is detectable in bulk samples, may go unnoticed when profiling a limited number of single cells. This is, however, not an artifact; rather it is a consequence of the small number of cells typically analyzed in single cell profiling studies, related to the highly skewed distribution of transcripts among individual cells due to transcriptional bursting, i.e. the large population of cells without or with very low transcription activity vs. a few cells exhibiting high expression^18^, and inherent limited sensitivity of the single cell RT-qPCR^19^,^20^.

### Requirements for high quality single cell RT-qPCR results

Even when analyzing single cells, the genomic DNA (gDNA), that is usually present in only two copies, can give rise to false positive results and compromise quantification. Conventional samples can be treated with DNase to remove gDNA^21^, but this is hard to include in a single cell workflow where the volume should be kept low, RNA concentration high, and washing steps avoided. The gDNA background can be measured and corrected by performing RT-minus controls, but this is not possible in single cell measurements because of the very small amount of material available. The general recommendation is to design the assays with the forward and reverse primers spanning an intron or to cross intron/exon junctions, which effectively eliminates amplification of the genomic copy^22^. However, not every gene has an intron and some have too short introns for this strategy to be efficient. In addition, many genes have pseudogenes that lack introns and are amplified^23^.

Some of the assays we designed had primer sets that do not span introns and are expected to amplify any gDNA present. These assays are indicated with red crosses in Fig. 1A. Assays without intron spanning primes are clearly off the fitted models. If considered, the fraction of positive cells would be seriously overestimated based on comparison to the bulk samples that were treated with DNase (Fig. 1A). This comparison is therefore the most effective method to assess the quality of single cell profiling data. Another quality control test in single cell RT-qPCR is to perform classical RT minus control reactions of the entire cell. Assays that produce positive signals evidently amplify gDNA and give rise to outliers in the average single-cell expression vs. bulk Cq value graph (Fig. 1B). These assays should be omitted from analysis.

### The sigmoidal fit enables normalization of the bulk Cq values similar to reference genes

**Figure 2:**
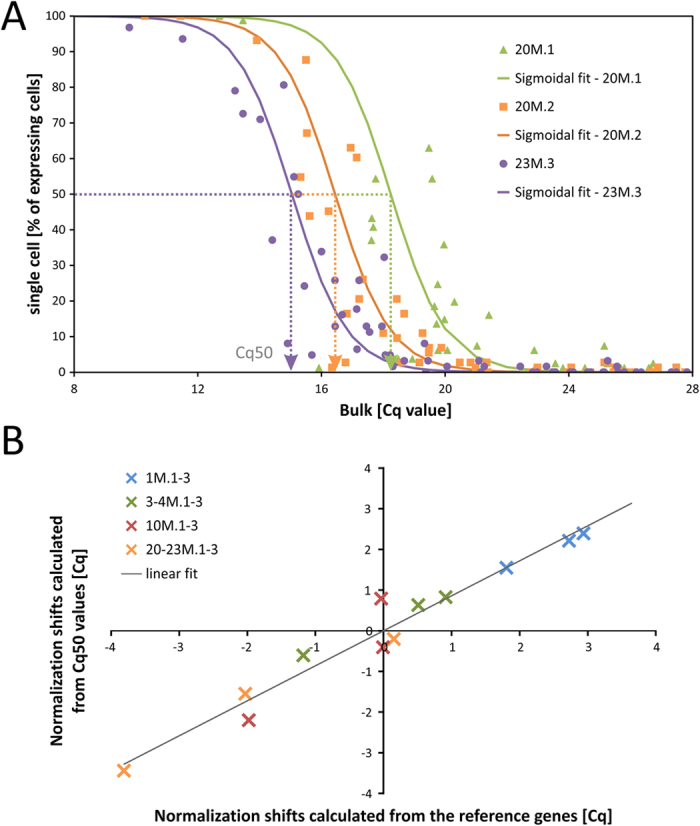
Normalization of bulk Cq values using single cell expression data. Normalization of bulk data (samples contained different number of cells) was performed using the standard approach based on the average expression of the most stable reference genes and using the parameter Cq50 expressing the bulk Cq value that corresponds to gene expression in 50% of the cells. The sigmoidal fit was calculated for each animal individually using the non-normalized bulk data. (**A**) Example of sigmoidal fits of data from three 20–23-month-old mice (20 M.1-3) with highlighted Cq50 values. Normalization was performed by shifting the Cq50 to reach the same value for each animal and keeping the average Cq50 of all animals unchanged at the same time. The same procedure was used for the average expression of reference genes. (**B**) Shifts of bulk data as calculated from *Cq50* values from sigmoidal fits and from reference genes in all 12 analyzed mice (1 M.1–23 M.3) plotted in the two-dimensional graph. High correlation (R^2^ = 0.957) confirmed the applicability of the normalization procedure based on the Cq50 parameter.

To test the robustness of the sigmoidal fit of the percentage of positive single cells to the bulk Cq values, the data from each of the 12 mice were analyzed separately (data from mice 20–23 M.1-3 are shown in Fig. 2A). This time the Cq values of the bulk samples were not normalized to the expression of reference genes, which spreads the data along the x-axis. Interestingly, differences between the calculated *Cq50* values corresponded with differences between Cq values of reference genes in these samples, which were used for normalization (see Supplementary Text S2). The normalization of bulk data performed using reference genes was therefore very similar to the normalization which would be performed according to the differences in calculated *Cq50* values (for comparison of normalization shifts see Fig. 2B). However, this could only be achieved with the gene sets which also included highly expressed genes, since these are needed in order to perform the sigmoidal fit correctly. Nonetheless, matching of the normalization shifts calculated from expression of reference genes, and from *Cq50* values, serve as an indirect validation of sigmoidal fitting.

Note also that normalization to the *Cq50* value does not require any reference genes to be known and can therefore be used to normalize bulk samples in situations when genes with stable expression cannot be identified, for example, when comparing different cell or tissue types. Another possible application could be the normalization of single cell RNA-Seq data, when commonly used methods failed due to the large proportion of zero values^24^. In such cases, an artificial bulk sample can be created by summing reads for each gene across pools of cells. Data in the log scale are plotted against the percentage of positive cells and fitted by the sigmoidal functions. Their parameters can be consequently used for normalization. However, the validity of this approach needs to be confirmed by other studies.

### The distribution of transcripts across cells

**Figure 3:**
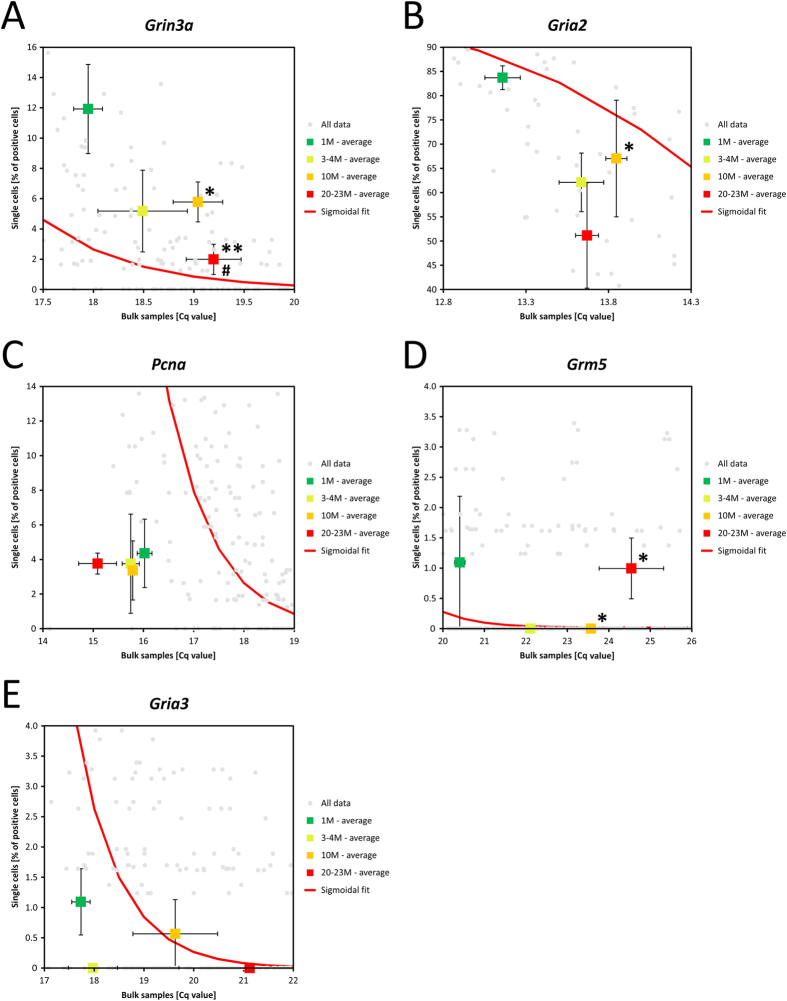
Single cell vs bulk expression 2D map. Plotting the single cells and bulk results in two-dimensional graphs provides a new valuable dimension to the interpretation of single cell expression data. The position of points relative to the sigmoidal fit indicates the expression level of a gene relative to the other genes. The changes in positions could reveal new biologically relevant information. Thanks to the two dimensions they could be recognized and statistically evaluated. Examples of the analysis for selected genes are provided: (**A**) *Grin3a*, (**B**) *Gria2*, (**C**) *Pcna*, (**D**) *Grm5* and (**E**) *Gria3*. Detailed description follows in the main text. Error bars indicate SEM. Significant differences between 1 M and 3–23 M (*p < 0.05, **p < 0.01) and between 10 M and 20–23 M (^#^p < 0.05), calculated with MANOVA.

The correlation between the expression of a particular gene measured in single cells, and its expression in corresponding bulk samples at different time points, can be shown in the plot of the fraction of positive single cells vs. bulk Cq values (Fig. 3). The fraction of positive cells and the Cq at each time point is indicated, including the standard error of the mean (SEM) with error bars. The sigmoidal fit of the fraction of positive cells vs. bulk Cq for all the genes is also shown. From this plot we can identify genes expressed at low level in a larger number of cells (positioned right from the sigmoidal fit, e.g. Fig. 3A) and genes expressed at high level in a smaller number of cells (positioned left from the sigmoidal fit, e.g. Fig. 3B,C), from cells with normal distribution of transcripts across cells as represented by the sigmoidal fit. This phenomenon cannot be seen when analyzing bulk samples, since only information about average expression can be extracted. The two-dimensional type of analysis also provides additional ways to identify significances that would go unnoticed in either bulk-based (p < 0.05 for 10 M vs. 20–23 M in Fig. 3A) or single cell (p < 0.05 for 1 M vs. 10 M in Fig. 3B) experiments only.

During the ageing process the fraction of cells expressing *Grin3a* decreases from about 12% at 1 M to 2% at 20–23 M, while Cq for the bulk samples increases from 18 to 19.2 cycles (Fig. 3A). Similar trend is seen for the highly expressed *Gria2* gene (Fig. 3B). Hence, for *Grin3a* and *Gria2,* the lower overall expression with ageing observed for the bulk sample is due to reduction of the number of cells expressing these genes. Another example of the importance of the position of measured data relative to the sigmoidal fit is *Pcna,* for which is the situation opposite (Fig. 3C). There is hardly any change in its expression during ageing as judged from the bulk Cq, and it is expressed in about 4% of the astrocytes independent of age (i.e. 1–2 cells per animal). The average number of *Pcna* transcripts in these cells must be much higher compared to other genes with similar expression, since the *Pcna* data are offset by almost 2 Cq values to lower values relative to the sigmoid fit. The total amount of *Pcna* transcripts in the 4% of the astrocytes that express *Pcna,* corresponds to the amount of transcripts typically found for other genes when expressed in 25% of the cells. This can be rationalized as the *Pcna* gene codes the proliferating cell nuclear antigen, which has high expression but only in actively proliferating cells^25^. Gene expression of *Pcna* in a very small fraction of astrocytes has been reported previously^26^.

Figure 3D,E show examples of genes with low expression, where single cell data in several cases fail to detect expression, while in the bulk samples expression of these genes is clearly seen to decrease during ageing. This provides evidence of the much higher sensitivity of bulk measurements compared to single cell profiling, which is particularly relevant for low expressed genes.

### The heterogeneity of GFAP/EGFP glial cells during aging

**Figure 4:**
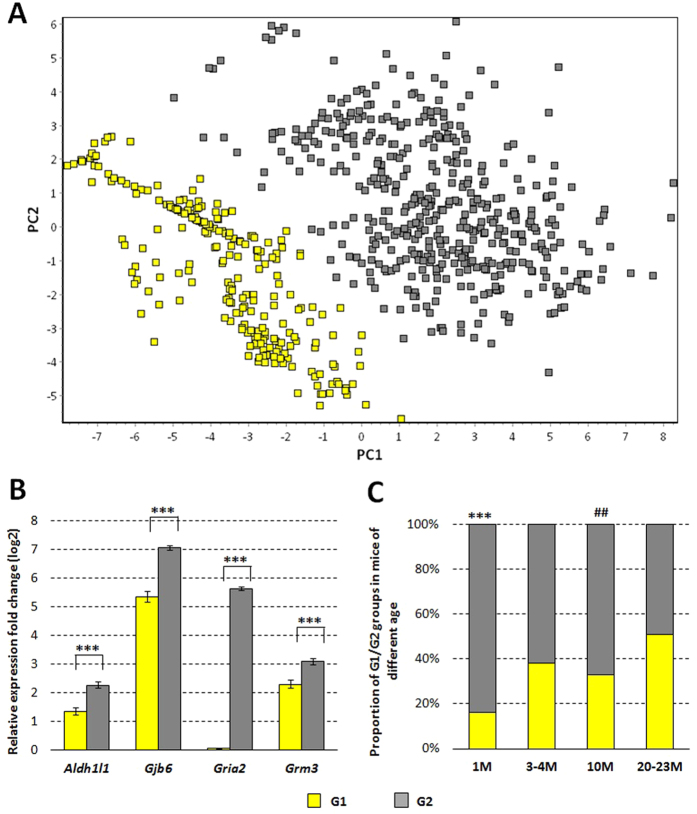
Clustering of GFAP/EGFP glial cells in two populations. (**A**) PCA analysis divided the cells from 1–23-month-old mice into two groups, G1 (yellow) and G2 (grey), based on differences in the gene expression. (**B**) The genes showing significantly different expression in the G1 and G2 groups. Error bars indicate SEM. Significant differences between G1 and G2 (***p < 0.001), calculated with Mann-Whitney test. For results with all tested genes, see Supplemental Text S3. (**C**) Proportion of G1 and G2 groups in mice of different age. Significant differences between 1 M and 3–23 M (***p < 0.001) and between 10 M and 20–23 M (^##^p < 0.01) calculated with Chi-squared test.

To explore the heterogeneity of GFAP/EGFP glial cells during aging, the complete data set (all single cells 1–23 M) was subjected to multivariate analysis. The principal component analysis (PCA) divided the cells into two groups, G1 and G2 (Fig. 4A), based on the difference in the gene expression - significant differences were observed in the expression of *Gria2, Gjb6, Aldh1l1* and *Grm3* genes (Fig. 4B). Intriguingly, the division into two clusters was influenced by the age of the animals the cells were isolated from – 16.3% and 50.7% of cells in G1 group in 1 M and 20–23 M cells, respectively (Fig. 4C). A more detailed analysis divided by the age groups is provided in the Supplementary Text S3 and the results show that the division of the cells into groups G1 and G2 (and corresponding gene expression profiles) were stable in all age groups. This analysis thus shows that GFAP/EGFP glial cells do not form a homogeneous glial cell group but part of these cells shows higher expression of genes coding glutamate receptors GluA2 and mGluR3 (*Gria2* and *Grm3*), gap beta-6 junction protein (*Gjb6*) and 10-formyltetrahydrofolate dehydrogenase (*Aldh1l1*) and these cells are more numerous in younger animals.

The largest impact on the division into G1 and G2 groups had *Gria2* gene (Fig. 4B), as this gene was highly expressed in G2 group and its expression in G1 group was at the limit of detection. *Gria2* gene is a gene coding GluA2 subunit of AMPA receptors, which are crucial receptors in glutamate signaling pathway. Presence of this subunit markedly diminishes Ca^2+^ permeability of AMPA receptors and for example cerebellar Bergmann glial cells were shown to lack this subunit^27^,^28^. In fact, this subunit is present in the most of the GFAP/EGFP glial cells and unlike other glutamate receptor subunits, its expression doesn’t even significantly change after middle cerebral artery occlusion (MCAO) model of ischemic injury^29^. In the mouse cortical region it is present also in neurons, oligodendrocyte progenitor cells (NG2-glia) and in newly formed oligodendrocytes^26^. The fact that there are two distinct subpopulations of GFAP/EGFP glial cells, which differ mainly in the presence or absence of *Gria2* gene expression, raises a question of functional consequences for these cells. From all the cells we analyzed (693 cells), 68% (471 cells) of them expressed at least one AMPA receptor subunit (*Gria1–4*) and the vast majority of these cells (96.5%, 455 cells) expressed *Gria2* gene. We can thus assume that in general, cells which did not express *Gria2* gene didn’t possess AMPA receptors and were thus not able to fully participate in glutamate signaling. Even though glutamate signaling in glial cells is still not completely elucidated, we hypothesize, that GFAP/EGFP glial cells, which possess AMPA receptors, express also *Gria2* gene to ensure limited Ca^2+^ influx and providing thus protection against glutamate excitotoxicity. Nonetheless, the validity of this conclusion needs to be confirmed by functional studies.

## Conclusions

The study has shown that single cell expression profiles measured with RT-qPCR, are fully consistent with the expression measurements in bulk samples, when the background due to gDNA is considered. Low level transcripts may go undetected in single cell studies, because of the small number of cells typically analyzed in an experiment, such that those transcripts are not present in any of the cells analyzed. Reliable measurement of the expression of such low expressed genes requires bulk samples. Finally, the combination of a single cell and bulk approach offers the possibility to validate the measured data and provides valuable biological insight.

## Methods

### Sample collection

All experiments were performed on cells from acutely isolated brains of GFAP/EGFP transgenic mice [line designation TgN(GFAPEGFP)]^16^. All procedures involving the use of laboratory animals were performed in accordance with the European Communities Council Directive 24 November 1986 (86/609/EEC) and animal care guidelines approved by the Institute of Experimental Medicine, Academy of Sciences of the Czech Republic (Animal Care Committee on April 17, 2009; approval number 036/2012).

The mice were anaesthetized, and cerebral cortices were removed and used for preparation of cell suspension using a papain dissociation kit (Worthington, NJ, USA). The astrocytes were collected using FACS (BD Influx, CA, USA), based on their EGFP fluorescence. Initially, cells were sorted into 96-well plates, each well containing 5 μl of nuclease-free water with bovine serum albumin (BSA, 1 mg/ml, Thermo Fisher Scientific, MA, USA). One cell was sorted into each well. The collection medium prevented unspecific RNA binding to plastic surface, enhanced reverse transcription efficiency and supported RNA stability^17^. Noteworthy, direct cell lysis with BSA was also proved to be superior to standard column based extraction methods^17^. After collecting 2–3 plates of single cells, remaining cells were collected as a bulk sample into an Eppendorf tube containing 500 μl of RLT-buffer with β-mercaptoethanol (RNeasy Micro Kit, Qiagen, Germany). Samples were immediately frozen to −80 °C and stored until analysis. The complete procedure has been published previously^30^. The detailed description can be found as Supplementary Text S4.

The cells were collected from a total of twelve 1–23-month-old GFAP/EGFP mice, with three at each time-point: 1-month-old (1 M.1-3), 3-4-month-old (3–4 M.1-3), 10-month-old (10 M.1-3) and 20–23-month-old mice (20–23 M.1-3). The number of cells collected and analyzed from each mouse is shown in Table 1.

### RT-qPCR and data analysis

RNA from the bulk samples was extracted using RNeasy Micro Kit (Qiagen) according to the manufacturer’s instructions, which included DNase treatment. Four μl of RNA were reverse transcribed in two 10-μl reactions into cDNA using the standard protocol of SuperScript III Reverse Transcriptase (Life Technologies, CA, USA). cDNA was diluted 2-times and 4 μl was used for pre-amplification. Pre-amplification was performed in two 40-μl reaction mixes, each containing 48 pairs of primers (for sequences and distribution into the two mixes, see Supplementary Table S1). The two pools of pre-amplified cDNA were mixed, diluted 5-times, and 2 μl was used for qPCR performed in the Biomark high throughput qPCR instrument (Fluidigm, CA, USA). The expression of 95 genes was measured in each sample.

RNA extraction from single cells was not needed, because the RNA was already released during the collection stage (astrocytes are lysed by the osmotic pressure created in 5 μl nuclease free water, supplemented with 1 mg/ml BSA). RNA was reverse transcribed into cDNA. Four μl of non-diluted cDNA was pre-amplified using the same experimental set-up as for the bulk samples. Two pools of pre-amplified cDNA were mixed, diluted 2.5-times, and analyzed in the Biomark high throughput qPCR instrument. Detailed protocols describing reverse transcription, pre-amplification and qPCR have been published previously^30^. In addition, all protocols and information can be found as Supplementary Text S4.

RT-qPCR data were processed and analyzed with GenEx software (Ver. 6.0.1.612, MultiD). Reactions generating melting curves with deviating melting temperature (Tm) or aberrant melt profiles were considered negative. Samples showing no sign of any of the tested genes were excluded from analysis. Bulk data from different mice were normalized by the average of the expression in logarithmic scale of the most stable reference genes^31^, as identified by the NormFinder algorithm^32^ (see Supplementary Text S2). The normalization was performed by a positive or negative shift of Cq values, according to the differences observed in reference genes, so the average of all Cq values did not change. The normalized data are therefore presented as Cq values and not as ΔCq.

Multivariate analysis were performed in GenEx software (Ver. 6.0.1.612, MultiD). All missing data, for each gene separately, were replaced with the highest Cq +2. The Cq data were, for each gene separately, converted into relative quantities expressed relative to the sample with the lowest expression (maximum Cq) and transformed into a logarithmic scale with base 2. Data was further mean-centered and analyzed by PCA, dendograms and Kohonen self-organizing maps (SOM).

### Statistics

The fitting of the sigmoidal function was performed utilizing the Total Least Squares method, and linear fit was performed using Deming regression. Significant differences between data obtained from mice of different ages were identified by Multivariate Analysis of Variance (MANOVA) using R software^29^, Mann-Whitney test using GenEx software (Ver. 6.0.1.612, MultiD) and Chi-squared test using SAS software (Ver. 9.2).

## Additional Information

**How to cite this article**: Dzamba, D. *et al.* The correlation between expression profiles measured in single cells and in traditional bulk samples. *Sci. Rep.* **6**, 37022; doi: 10.1038/srep37022 (2016).

**Publisher's note**: Springer Nature remains neutral with regard to jurisdictional claims in published maps and institutional affiliations.

## Supplementary Material

Supplementary Information

## Figures and Tables

**Table 1:** Number of cells analyzed in each mouse (1 M.1–23 M.3).

mouse	1 M.1	1 M.2	1 M.3	3 M.1	4 M.2	4 M.3	10 M.1	10 M.2	10 M.3	20 M.1	20 M.2	23 M.3
single cells	61	62	61	53	32	38	51	59	60	81	73	62
bulk (thousands)	30	50	40	14.4	17.4	19.9	11.2	2.4	13.1	1.5	5	11.7

The second row indicates the number of analyzed single cells, and the third row indicates the number of cells in the bulk samples.
