# Supplementary material for: The correlation between expression profiles measured in single cells and in traditional bulk samples
Source: Sci Rep. 2016 Nov 16;6:37022. doi: 10.1038/srep37022 (PMC5111061; doi:10.1038/srep37022)
Supplement: Supplementary Information [file srep37022-s1.pdf]

Supplementary Information

The correlation between expression profiles measured in single cells and in traditional bulk samples

David Dzamba[1,2], Lukas Valihrach[3], Mikael Kubista[3], Miroslava Anderova[1,2*]

[1] Department of Cellular Neurophysiology, Institute of Experimental Medicine, Academy of Sciences of the Czech Republic, Prague, Czech Republic

[2] 2nd Faculty of Medicine, Charles University, Prague, Czech Republic

[3] Laboratory of Gene Expression, Institute of Biotechnology, Academy of Sciences of the Czech Republic, Vestec, Czech Republic

Corresponding author*

E-mail: anderova@biomed.cas.cz,

Tel.: +420 241 062 050, Fax: +420 241 062 782,

Current Address: Miroslava Anderova, Department of Cellular Neurophysiology, Institute of Experimental Medicine, AS CR, Videnska 1083, Prague 4, Czech Republic

Supplementary Table S1

Sequences of primers used in RT-qPCR

Gene Classification		Gene Name	NCBI RefSeq	Forward (5'-3')	Reverse (5'-3')	Size (bp)	Intron	Effeciency*	preAMP Mix**	References
Markers	Astrocytes	Slc1a3	NM_148938.3	ATCGTCCTGCCTCTCCTCTAC	GTCCACACCATTGTTCTCTTCCA	159	yes	0.98	M1	1
		Slc1a2	NM_011393.2	GAGAGCAGGCACACTTACA	GTAGAAGAATCTGGATACCGAAGG	159	no	0.92	M1	3
		Glul	NM_008131.3	CGCAAAGACCCCAACAAG	ATTCCTGCTCCATTCCAAAC	135	yes	0.96	M1	1
		Gfap	NM_001131020.1	ATGTGTCTCAGTTGTGAAGGTCTA	TGGAAGGATGGTTGTGGATTCT	108	no	1.00	M2	1
		Gfap delta	NM_010277.3	ACAGACTTTCTCCAACCTCCA	CAGGGCTCCATTTTCAATC	159	yes	1.00	M2	1
		S100b	NM_009115.3	TCTAACTCAGGACCGAGAATCA	GGAGCAAGGAAGATACAACTAACT	131	no	0.94	M1	1
		Aldh1l1	NM_027406.1	CTGGAAGATGGCAAGATGATG	TTCTACCTCTGGGACATTGG	147	yes	0.99	M1	1
	NG2	Cspg4	NM_139001.2	TGATGGAAGTGAGACACAGACAGA	GGAAGGATGGTGATCGTGAAGG	102	no	0.92	M1	1
		Pdgfra	NM_001083316.1	AAGAGACCCTCCTTCTACCAC	TATCAGAGTCCACCCGCAT	142	yes	0.97	M2	1
	Neurones	Rbfox3	NM_001039167.1	GGTGCTGAGATTTATGGAGGC	ATGGTTCCGATGCTGTAGGT	158	yes	0.92	M1	-
	Proliferation	Mki67	NM_001081117.2	CTCTGGCCTACCTGGTCTTA	TTCCTCTTGGTTGGCGTTTC	186	yes	0.97	M1	-
		Pcna	NM_011045.2	AATGTGGATAAAGAAGAGGAGGC	TGTAGGAGACAGTGGAGTGGC	114	yes	0.94	M1	-
	Intermediate filaments	Nes	NM_016701.3	AGCAACTGGCACACCTCAA	GGTATTAGGCAAGGGGGAAG	233	yes	1.00	M2	1
		Vim	NM_011701.4	TGCCAACCTTTTCTTCCCTG	TCTCTGGTCTCAACCGTCTT	109	yes	0.94	M2	1
Glutamate receptors	Kainate	Grik1	NM_146072.4	CACGAGACGGCTGCTGAA	ACCACTGTACCTGTAGAGTTCCA	126	yes	0.89	M1	1
		Grik2	NM_001111268.1	ACAATCAACAGGAACAGGACTCT	TGCTGATGAACTGTGTGAAGGA	159	yes	0.92	M1	1
		Grik3	NM_001081097.2	GCTCAGAGGTGGTGGAGAATA	GCCGTGTAGGAGGAGATGAT	166	yes	0.96	M1	1
		Grik4	NM_175481.5	CGCATGGTAGAATTGGAAGGT	AAGAGACTGTCAGAGATGTTGGA	188	yes	0.90	M1	1
		Grik5	NM_008168.2	CCACCTTGTCCTCCGTAA	CTCCACGATACCATCCAGAT	109	yes	0.89	M1	1
	AMPA	Gria1	NM_001113325.1	ACTCAAGCGTCCAGAATAGAAC	AATCTCAAGTCGGTAGGAATAGC	173	yes	0.94	M1	1
		Gria2	NM_001039195.1	CAGATTGTAGACTACGACGACTC	TCATCACTTGGACAGCATCATAA	175	yes	0.96	M1	1
		Gria3	NM_016886.3	GGTCATTCTCACGGAGGATTC	GGTGTTCTGGTTGGTGTTGTA	123	yes	1.00	M1	1
		Gria4	NM_001113180.1	CCAGTAGAGGACAACGCAATT	TGACAGAGTGAAGGTTACAGGAA	155	no	0.91	M1	1
	NMDA	Grin1	NM_001177656.1	AGATAGTGACAATCCACCAAGAAC	ACCATTGACTGTGAACTCCTC	89	no	0.91	M1	1
		Grin2a	NM_008170.2	GACCAGATGCTTCAGGAGACAG	CTTGAGGCTTATGCTACGAGAGG	192	no	0.97	M1	1
		Grin2b	NM_008171.3	GGTGTTTAACAACTCCGTACCT	GAAACCTGGTCCACATACTCCTC	156	no	0.95	M1	1
		Grin2c	NM_010350.2	CGTGTGGTTAGTACCTAATCTG	TTCTGGCGTAGGCTAAGG	114	no	0.95	M1	1
		Grin2d	NM_008172.2	AACCGAGACTACTCCTTCAATGA	GCCATAGCGGGACCATAGA	150	yes	0.95	M1	1
		Grin3a	NM_001033351.1	GACAAAGCCCTTCTGGATTATGA	ATGTTAGAGGTCAACGGAGAGT	128	yes	0.91	M1	1
		Grin3b	NM_130455.2	TGGTCGGGGACAAGACATTT	TCCGTGTGGAGTGGTAGGTG	182	yes	0.94	M1	-
	Metabotropic	Grm1	NM_001114333.2	CGAGTGGAGTGACATAGAATC	TACCAGCCAGAATGATATAGCA	155	no	0.88	M1	1
		Grm2	NM_001160353.1	GCTCCTACAGTGATGTCTCCA	CGAGCAAAGTAATCATAACGGG	124	yes	0.92	M1	-
		Grm3	NM_181850.2	CGACCACATATTCTCAGTCCTCT	AGCACTTCGTCTAACAGCCTATA	140	yes	0.96	M1	1
		Grm4	NM_001013385.1	ACCAAGCCTGAACGAGTG	CGGGAGAAGAAGTCATAGCG	158	yes	0.98	M1	-
		Grm5	NM_001081414.2	CAGCTTAGATCGCAGCCACT	CAAGAATTTGGGTAAAATCACCA	133	yes	0.91	M1	1
		Grm6	NM_173372.2	CAACTATGGTGAAAGCGGGG	TTGGGCAATACAGACACCTC	73	yes	0.99	M1	-
		Grm7	NM_177328.3	AGCCATCACCATCCAACCT	TCGGCAAACCATACATTTCTCC	102	yes	0.92	M1	-
		Grm8	NM_008174.2	CAGCAAGCTCCGTGTCCATC	GACCACCCGAGAAAAGAAATCA	128	yes	0.94	M1	-
Other receptors	Purinergic	P2rx1	NM_008771.3	TGGGAGTCATTTTCCGTCTG	GCCACTTGAGGTCTGGTATC	95	yes	0.94	M1	-
		P2rx2	NM_001164833.1	TAGAGCAAGCAGGAGAGAACT	CTGAAGAGGCAGGGTCATACT	159	yes	0.93	M1	-
		P2rx5	NM_033321.3	AGCAAAGTTCGTGGTCGC	AAGCATCGTGGTGTTGGTAT	190	yes	0.96	M1	-
		P2ry4	NM_020621.4	AAGGTTCCTAGGCAAGGGT	CTGAAGAGGGTGGAGGGTAA	132	yes	0.97	M1	-
		P2ry6	NM_183168.2	ACCCAACCTGCCTTGAAAAC	ATTGTCCTGCTCCATAACTGC	129	yes	0.94	M1	-
	Endocannnabinoid	Cnr1	NM_007726.3	AGCTCTCATAGAGTCTGGGG	GTGATGGTACGGAAGGTGGT	221	yes	1.00	M1	-
	Thrombin	F2r	NM_010169.3	TTCCCGCGTCCCTATGAG	CCAGGGGGACCAGTTCAAAT	113	yes	0.97	M1	-
Channels	Potassium (TREKs)	Kcnk2	NM_010607.2	ATTCGTATCATCTCCACCATCATC	CACACCACAGGCTTGTAGAA	209	yes	0.84	M2	1
		Kcnk10	NM_029911.4	CACTGTGGCTATCTCCTTAACC	GGCTGAGGCGGTGTAATC	111	no	0.95	M2	1
		Kcnk1	NM_008430.2	GGGAAATTGGAATTGGGACTTCA	TGCCGATGACAGAGTAGATGAT	134	yes	0.97	M2	1
		Kcnk3	NM_010608.2	CCAGCCTTCCACTACCTTAC	CACACTCTGCTCTAACATCTGT	200	no	0.99	M2	-
		Kcnk5	NM_021542.4	ACCCGCTCATCTTCCAAAAT	ATGAAGGGAATTCACCCGTG	171	no	1.00	M2	-
		Kcnk9	NM_001033876.1	TGACTACTATAGGGTTCGGC	GAATCGCAGGACCACAAGAT	149	no	0.94	M2	-
	Chloride	Clcn1	NM_013491.2	GCAGAGCGAAGGTTGAAGG	TCTACGAAGGCAAAGGACTCAG	128	no	0.90	M2	2
		Clcn2	NM_009900.2	TGCCAATGTCTTCCTTACTCTG	ATTCGGTAGGTGCTGCTATC	198	yes	0.96	M2	1
		Clcn3	NM_007711.3	AATTGTTGACGATATTCCTGACC	TGGGGATAAACAAGCCTGAC	151	yes	0.96	M2	3
		Clcn4	NM_011334.3	CCTCGTGCTCGTCGTATC	CCTCTTGCCTTCATTCTTGCTATA	127	no	0.98	M2	3
		Clcn7	NM_011930.3	CATTGACATTGTAGTGGAGAACCT	CACGAGTACGAAGGCAGAG	139	yes	0.98	M2	3
		Vdac1	NM_011694.4	TCGCAGTTGGCTATAAGACG	TTCCGAAGCGAGTGTTACTG	156	yes	0.95	M2	3
		Vdac2	NM_011695.2	CTTACACTGTCTGCTCTGGTAG	GCCAAATCTTCTGATATTCGTTCC	129	no	0.94	M2	3
		Vdac3	NM_011696.1	TGGGAGAATAAGTTGGCTGAAG	TACTGCCGAGACTAAAACAATCC	124	yes	0.96	M2	3
		Best1	NM_011913.2	GGCTCCACCTTCAACATCAG	GCCACTCTCTTTCTTATCCGT	87	yes	1.03	M2	-
	HCN	Hcn1	NM_010408.3	CTCAGTCTCTTGCGGTTATTACG	TGGCGAGGTCATAGGTCAT	91	yes	0.97	M2	1
		Hcn2	NM_008226.2	ATCGCATAGGCAAGAAGAACTC	CAATCTCCTGGATGATGGCATT	102	yes	0.96	M2	1
		Hcn3	NM_008227.1	GATGTTTGATGAAGAGAGCATCC	CCCGGCAGGTGAAGTTAATA	77	yes	0.96	M2	1
		Hcn4	NM_001081192.1	GCATGATGCTTCTGCTGTGT	GCTTCCCCCAGGAGTTATTC	123	yes	0.93	M2	1
	TRPV	Trpv4	NM_022017.3	AGAGACAAGTGGCGTAAGTT	GCTGATAGTAGGCGGTGAG	100	no	0.94	M2	1
	AQP	Aqp1	NM_007472.2	CTGGCTGCGGTATCAACC	GGATGAAGTCATAGATGAGCACTG	132	yes	0.96	M2	1
		Aqp4	NM_009700.2	CGGCATCCTCTACCTGGTCACA	GCCAGCGGTGAGGTTTCCAT	82	yes	0.96	M2	1
		Aqp9	NM_022026.2	GAAGGATGGAGTGGTTCAAGTTC	TGGCACGGATACAAATGGTTT	137	yes	1.00	M2	1
Transporters	connexin	Gja1	NM_010288.3	TCTCACCTATGTCTCCTCCT	GCTGGCTTGCTTGTTGTA	86	no	0.99	M2	2
		Gjb6	NM_001010937.1	TAAGAATAAGCCTGCACGATGGA	CCACTAGGATCATGACTCGGA	130	no	0.93	M2	2
	co-transporters	Nkcc1	NM_009194.3	ATTACGGTGGCTAACACTGG	GCATCTCTAAGAACTAATGGTTGA	98	no	0.94	M2	3
		Kcc1	NM_009195.2	TACAAGTACATCGAGTACCAAGG	GTCTAACTTAAGCAGCACCAGGAG	159	yes	0.95	M2	3
		Kcc3	NM_133648.2	CATTCCAGGGTTGGCTAGTG	TGTGACCGAGGGAAAGAAGA	190	yes	0.92	M2	2
	Glucose transporter	Slc2a1	NM_011400.3	ACATGGAACCACCGCTACG	CCGACAGAGAAGGAACCAATCAT	113	no	0.96	M2	-
	Lactate trasporters	Slc16a1	NM_009196.3	AAGTCTCTGTTGATGGTGGAAT	GAGTGTTGGTGACCCTGTATC	127	no	0.95	M2	-
		Slc16a3	NM_001038653.1	TTCTCCAGTGCCATTGGTCTC	CCGCCAGGATGAACACATACTT	121	yes	0.93	M2	-
Snare complex and others	Synaptosomal-associated protein	Snap25	NM_011428.3	GCAATAATCAGGATGGAGTAGTGG	TGGCGATTCTGGGTGTCAAT	214	yes	0.93	M1	1
		Snap23	NM_001177793.1	GGAGTATCCGTATTTCTGTGCC	TGCGATTTAGTTGTTCCCCTT	205	yes	0.95	M1	-
	Vesicle-associated proteins	Vamp2	NM_009497.3	TGCACCTCCTCCAAACCTTA	CCTTGTCCACATTCACCCTC	98	yes	0.95	M1	-
	Synaptotagmins	Syn12	NM_134164.5	CTGACTGTGGTTGTGGTGAAA	AATGGCTGGCACTGAGAAGAT	186	yes	0.94	M1	-
	Syntaxins	Stx18	NM_026959.2	CTTGTTTGACGAAGTGAGGC	CGACTAACTGGTGAATGCTGT	122	yes	0.94	M1	-
	Vesicular glutamate	Slc17a7	NM_182993.2	TGACCATCATCGTACCCATTG	GAGTATCCGACCACCAGCAG	142	yes	0.94	M1	-
		Slc17a6	NM_080853.3	GCTTATTTTGAGGAGGTTTTTGGAT	ATGACAAGGTGAGGGACTG	74	yes	0.94	M1	-
	Vesicular ATP transporter	Slc17a9	NM_183161.3	AGGGTTACAGAGTCATCACGG	CAGGGCAAAAATGCTTGACAG	74	yes	0.92	M1	-
Reference genes		Actb	NM_007393.4	assay provided by commercial vendor, sequence unknown		94	yes	>0.9	M2	4
		Arbp	NM_007475.5	assay provided by commercial vendor, sequence unknown		235	yes	>0.9	M2	4
		B2m	NM_009735.3	assay provided by commercial vendor, sequence unknown		107	yes	>0.9	M2	4
		Gapdh	NM_001289726.1	assay provided by commercial vendor, sequence unknown		109	yes	>0.9	M2	4
		Gusb	NM_010368.1	assay provided by commercial vendor, sequence unknown		169	yes	>0.9	M2	4
		Hprt1	NM_013556.2	assay provided by commercial vendor, sequence unknown		141	yes	>0.9	M2	4
		Pgk1	NM_008828.3	assay provided by commercial vendor, sequence unknown		170	yes	>0.9	M2	4
		Ppia	NM_008907.1	assay provided by commercial vendor, sequence unknown		128	yes	>0.9	M2	4
		Tbp	NM_013684.3	assay provided by commercial vendor, sequence unknown		195	yes	>0.9	M2	4
		Tubb	NM_009451.3	assay provided by commercial vendor, sequence unknown		238	yes	>0.9	M2	4
		Ywhaz	NM_001253805.1	assay provided by commercial vendor, sequence unknown		222	yes	>0.9	M2	4

*amplification efficiency of a qPCR reaction based on the slope of the standard curve calculated using the formula Efficiency = -1+10^(-1/slope)

** distribution of primers into two pre-amplification mixes containing different sets of primers (preAMP mixes M1 and M2); details concerning the pre-amplification procedure are provided in Supplemental Text 1

[1]Rusnakova *et al*. PLoS One. 2013;8(8):e69734. doi: 10.1371/journal.pone.0069734.

[2]Anderova *et al*. PLoS One. 2014;9(11):e113444. doi: 10.1371/journal.pone.0113444.

[3]Benesova *et al*. PLoS One. 2012;7(1):e29725. doi: 10.1371/journal.pone.0029725.

[4]Reference Gene Panel Mouse (A102; Tataa Biocenter)

Supplementary Text S2

Reference gene selection

A

B

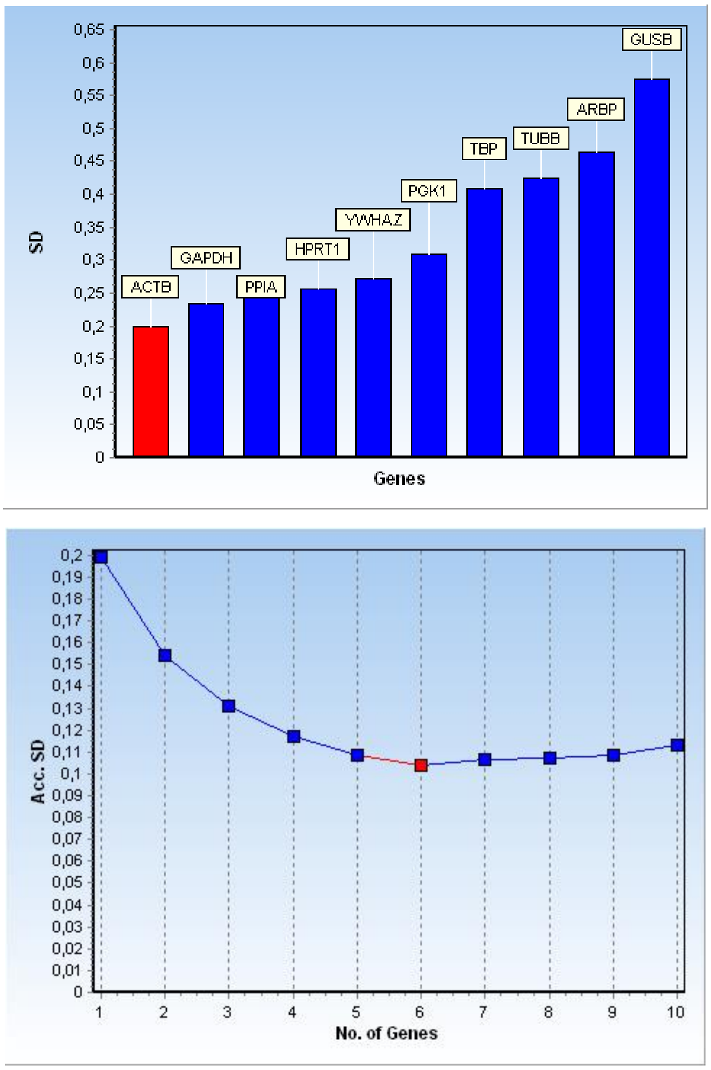

Fig. S1. Reference gene selection. The stability of 10 reference genes was evaluated by NormFinder algorithm based on authors recommendations (Andersen *et al*. Cancer Res. 2004;64(15):5245-50). **(A)** NormFinder ranks the set of candidate normalization genes according to their expression stability (expressed as standard deviation SD) in a given sample set and given experimental design. The lower value indicates higher stability. **(B)** NormFinder also determined the optimal number of reference genes based on the accumulated standard deviation (Acc. SD). The lowest value was achieved by combination of 6 reference genes (*Actb*, *Gapdh*, *Ppia*, *Hprt1*, *Ywhaz*, *Pgk1*), which were used for normalization of bulk data. The optimal number of reference genes is indicated by the red square. Visualization was performed by GenEx software (MultiD), which includes the NormFinder algorithm.

Supplementary Text S3

Multivariate analysis of experimental data

Data was preprocessed as described in the manuscript. To be able perform multivariate testing, missing values were replaced with the highest Cq +2 (for each gene separately). The data were converted into relative quantities expressed relative to the sample with the lowest expression (maximum Cq) and transformed into a logarithmic scale with base 2. Data was mean-centered and used for analysis using basic multivariate analysis method as principal component analysis (PCA), dendograms and Kohonen self-organizing maps (SOM). Only data passing the quality control based on Fig. 1 in the manuscript was included in the analysis (i.e. assays generating signals in RT- controls or assays which primers that do not span introns were excluded from the analysis). In the case of multivariate analysis, if a gene did not pass the control for any sample, complete data set for the given gene was excluded. The first analysis was performed using the bulk data (Fig. S2-4), following by single cell data (Fig. S5-7). All analysis and visualization were performed in the GenEx software (Ver. 6.0.1.612, MultiD).

Bulk data analysis

Different multivariate analysis methods were applied on the experimental data set containing only bulk data (Fig. S2-4). Data was normalized using the average of six reference genes (Supplementary Text S2). Data was analyzed as individual samples as well as the average of samples of the same age. As expected, dendograms, PCA and SOM clustered samples into distinct groups based on their age. The most different groups were 1M and 20-23M animals, whereas animals from 3-4M and 10M were the most similar ones and in several analyses created mixed groups. If the same analyses were performed on averaged data based on the age of animals, the clustering was more efficient clearly dividing groups of differently old animals.

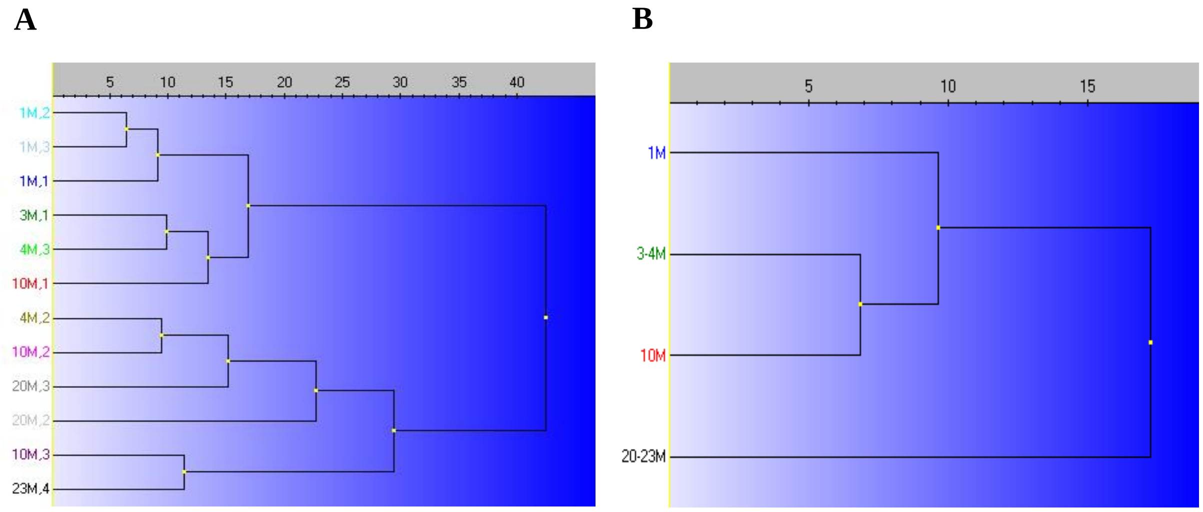

Fig. S2. Hierarchical clustering of bulk data using dendograms. Ward's algorithm and Euclidean distance measurement was used to generate dendograms. Samples formed clusters based on the age of animals. (**A**) Dendogram of individual samples. (**B**) Dendogram based on different age of animals.

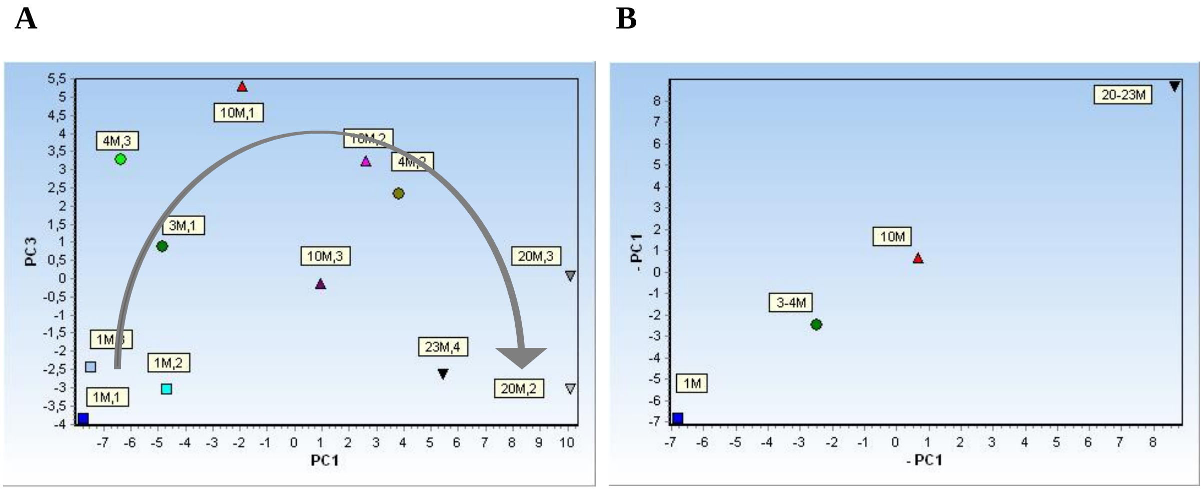

Fig. S3. Principal component analysis of bulk data. Samples formed clusters based on the age of animals. (**A**) PCA of individual samples. The arrow indicate the direction of development. (**B**) PCA based on different age of animals.

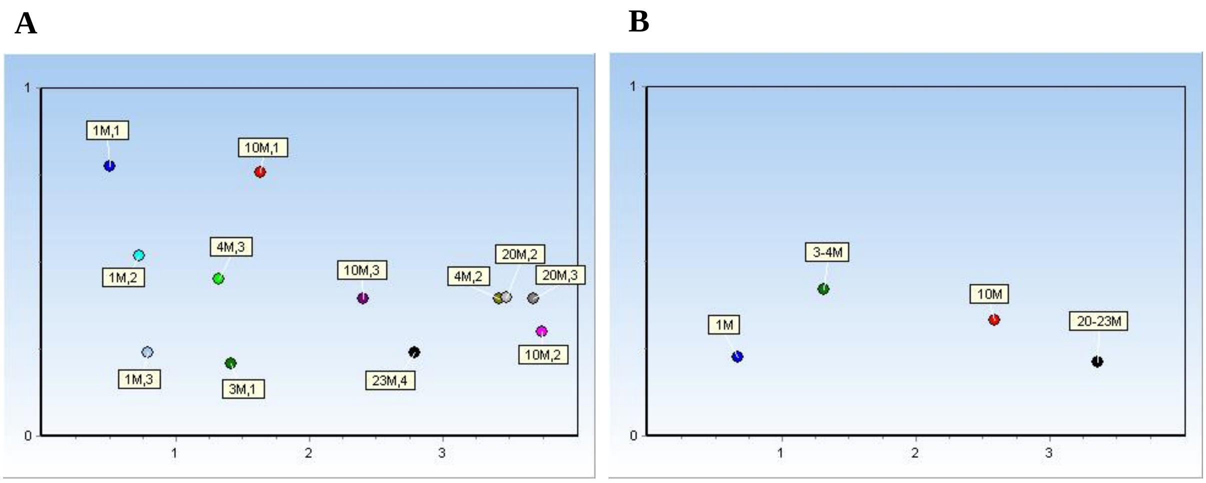

Fig. S4. Kohonen self-organizing maps of bulk data. Samples were distributed into groups based on their similarity. (**A**) SOM of individual samples. (**B**) SOM based on different age of animals.

Single cell data analysis

Single cell data was analyzed in a similar way as the bulk data. As was already described, PCA analysis identified two groups of cells, G1 and G2, which significantly differed in the expression of several genes (for details see the main text of this manuscript and Fig. 4). The both groups contained cells from all animals (Fig. S5-6). The division of cells into two groups was stable in all age groups (Fig. S7). The analysis revealed that cells do not form a homogeneous population but part of these cells shows different expression pattern. It is important to note, that the pattern of the genes which divided the cells into two groups was conserved across the age groups (*Gria2, Gjb6, Aldh1l1* and *Grm3* genes being the most important). As the most differently expressed gene was identified *Gria2* gene. The biological consequences of this finding are discussed in the main text of the manuscript.

A

B

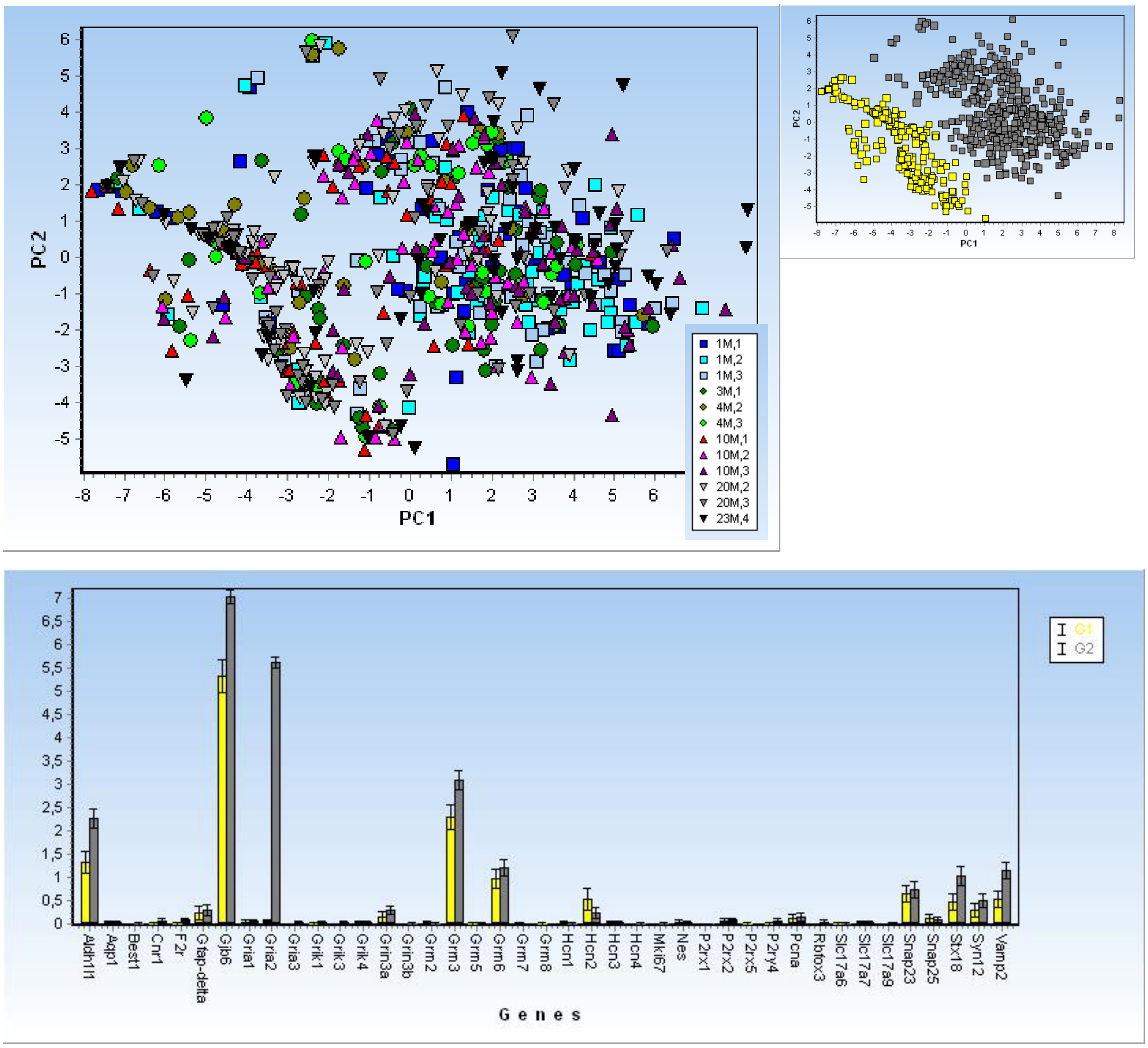

Fig. S5. Principal component analysis of single cell samples. (**A**) Single cells from different animals are depicted by various symbols (top left). Cells were divided into two groups (top right) - designed G1 (yellow symbols) and G2 (grey symbols) – top right. (**B**) The expression of tested genes in G1 and G2.

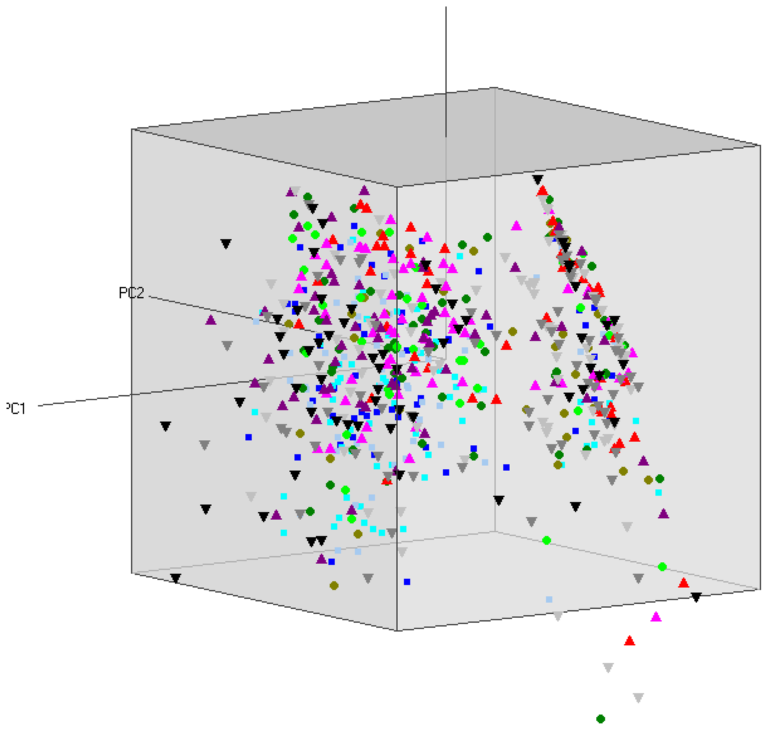

Fig. S6. Principal component analysis of single cell samples in 3D. Single cells from different animals are depicted by various symbols.

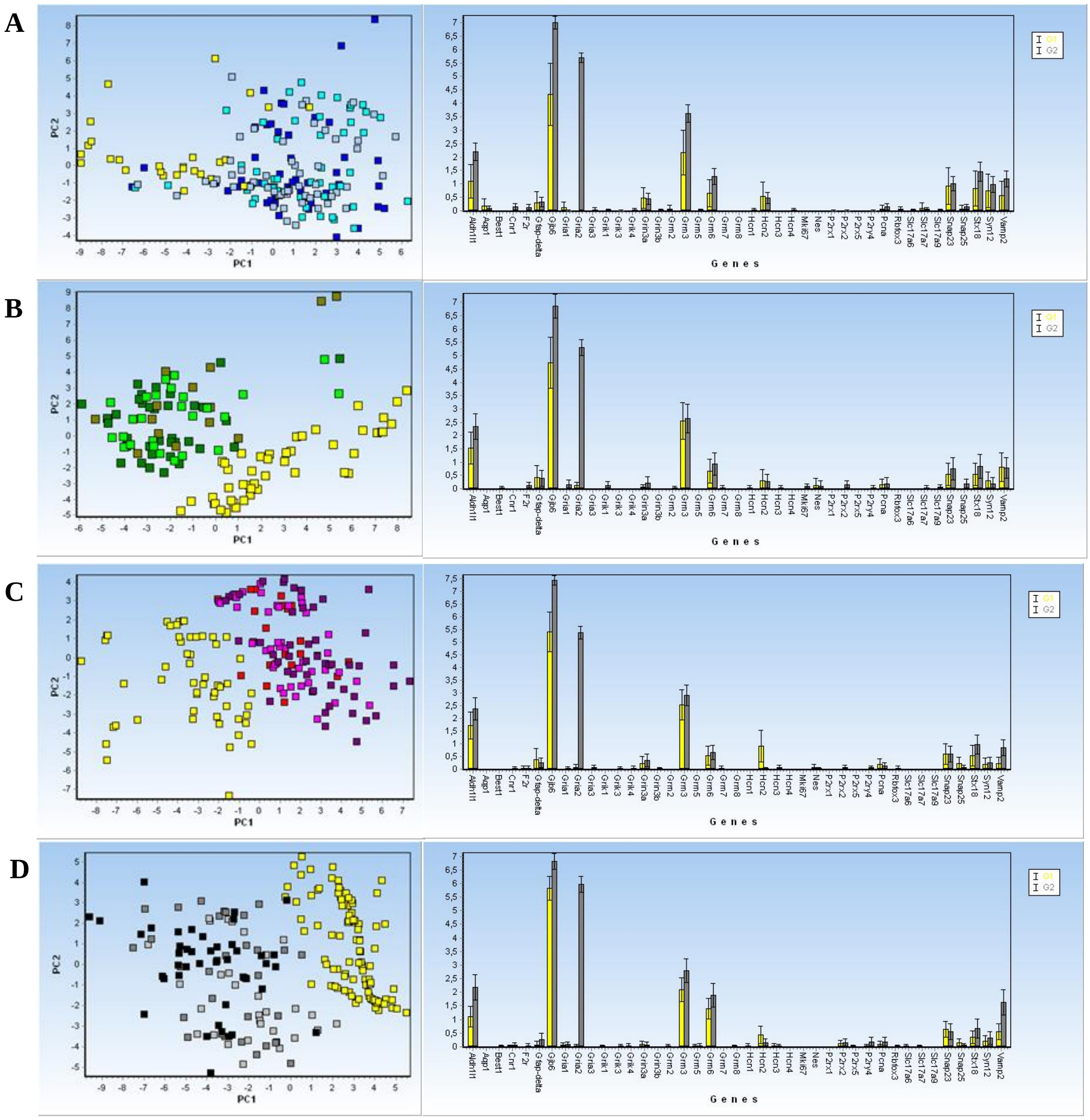

Fig. S7. Principal component analysis of single cell samples in different age groups: (A) 1M, (B) 3-4M, (C) 10M and (D) 20-23M. Single cells from different animals are depicted by various colors. The G1 group is highlighted by yellow. Each principal component analysis (left) is supplemented by the graph showing the expression of genes in G1 and G2 in the given age group (right).

Supplementary Text S4

MIQE

Minimum Information for Publication of Quantitative Real-Time PCR Experiments

Information designed according to the MIQE guidelines as essential (E) is presented, desirable information (D) attached if available.

EXPERIMENTAL DESIGN

Definition of experimental and control groups (E)

The expression of 95 genes was measured using RT-qPCR technique in cortical astrocytes isolated from 1-, 3-4- , 10- and 20-22-month-old GFAP/EGFP mice (4 groups). Single astrocytes as well as bulk astrocyte samples were collected.

Number within each group (E)

Three mice at each time point: 1M.1-3, 3M.1-3, 10M.1-3 and 20M.1-3. The number of cells collected and analyzed from each mouse is shown in Table 1 in the manuscript.

Assay carried out by core lab or investigator's lab? (D)

Assay was carried out by investigator's lab.

SAMPLE

Description (E)

All experiments were performed on cells from acutely isolated brains of GFAP/EGFP transgenic mice [line designation TgN(GFAPEGFP)], in which the expression of enhanced green fluorescent protein (EGFP) is controlled by the human promoter for glial fibrillary acidic protein (GFAP). The astrocytes were collected using FACS (BD Influx, CA, USA), based on their EGFP fluorescence.

Volume/mass of sample processed (D)

The number of cells collected and analyzed from each mouse is shown in Table 1 in the manuscript.

Microdissection or macrodissection (E)

not applied

Processing procedure (E)

The mice were deeply anaesthetized with pentobarbital (PTB, 100 mg/kg, i.p.) and perfused transcardially with cold (4–8°C) isolation buffer containing (in mM): NaCl 136.0, KCl 5.4,

Hepes 10.0, glucose 5.5, osmolarity 290±3 mOsmol/kg. The forebrain was isolated by the removal of the olfactory lobes, cerebellum, and midbrain/hindbrain structures by dissection. To isolate the cerebral cortex, the brain (+2 mm to -2 mm from bregma) was sliced into 400 µm coronal sections using a vibrating microtome HM650V (MICROM International GmbH), and the dorsal cerebral cortex was carefully dissected out from the ventral white matter tracks. The tissue was incubated with continuous shaking at 37°C for 90 minutes in 5 ml of a papain solution (20 U/ml) and 0.2 ml DNase (both from Worthington) prepared in isolation buffer. After papain treatment, the tissue was mechanically dissociated by gentle trituration using a 1 ml pipette. Dissociated cells were layered on the top of 5 ml of Ovomucoid inhibitor solution (Worthington) and harvested by centrifugation (140xg for 6 minutes). This method routinely yielded, ~$2x10^6$ cells per mouse. Cell aggregates were removed by filtering with a 30 µm cell strainer (Becton Dickinson), and the cells were kept on ice until sorting. Single cells were sorted using flow cytometry (BD Influx). The flow cytometer was manually calibrated to deposit a single cell in the center of each collection tube. Hoechst 33258 (Life Technologies) was added to the suspension of cells to check viability. Initially, cell sorting was performed with a negative control (no fluorescent cells) in order to set the fluorescence threshold for collecting EGFP+ cells and to avoid auto-fluorescent cells. After setting the threshold, all EGFP+ cells that crossed this level of fluorescence were collected (Fig. S8).

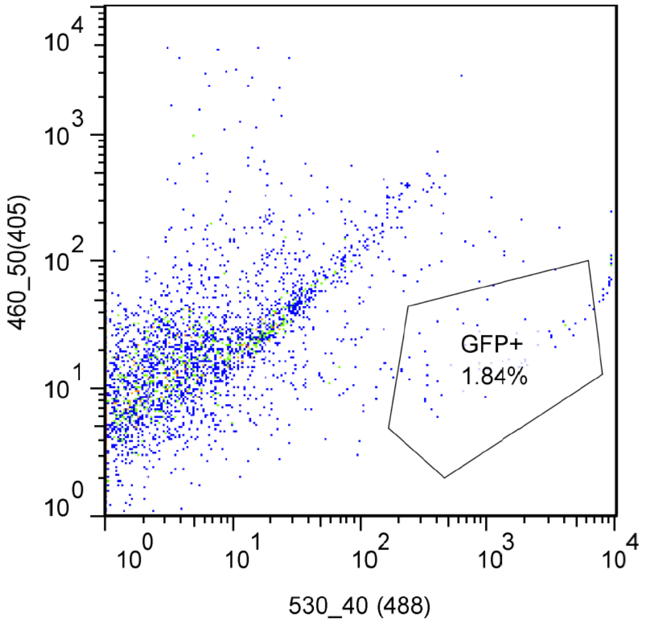

Fig. S8: An example of the FACS plot from cell collections. On the x-axis there is EGFP fluorescence (488 nm) and on the y-axis there is Hoechst fluorescence (405 nm) plotted. The

sorting gate was set relatively strict (marked by a pentagon within the plot), so only ca. 1.2-3% of all cells were collected. The collected cells had high EGFP and low Hoechst fluorescence.

Single cells were collected into 96-well plates (Life Technologies) containing 5 µl nuclease-free water with bovine serum albumin (1 mg/ml, Invitrogen). Plates were placed on a pre-cooled rack. The cells collected were positive for EGFP and viable. Collected cells in 96-well plates were immediately placed on dry ice. After collecting 2-3 plates of single cells, remaining cells were collected as a bulk sample into an Eppendorf tube containing 500 µl of RTL-buffer with β-mercaptoethanol (RNeasy Micro Kit, Qiagen). Samples were immediately frozen on dry ice and stored at -80°C until analysis. The homogeneity of the sorted cell population was confirmed by subsequent single cell analysis using groups of cell-specific markers (see Supplementary Table S1).

If frozen - how and how quickly? (E)
After each plate or bulk sample was sorted, it were immediately frozen on dry ice and stored at -80°C until analysis.
If fixed, with what and how quickly? (E)
not applied
Sample storage conditions and duration (especially for FFPE samples) (E)
Samples were stored at -80 °C till RNA extraction (no longer than 1 months).

NUCLEIC ACID EXTRACTION
Procedure and/or instrumentation (E)
RNA from bulk samples was extracted using RNeasy Micro Kit (Qiagen) according to the manufacturer's instructions. RNA from single cells was released into 5 µl NFW supplemented by BSA (1 mg/ml) immediately after sorting process (osmotic lysis).
Name of kit and details of any modifications (E)
RNeasy Micro Kit (Qiagen) - protocol involved usage of RNA carrier and DNase treatment (both contained in the kit); no modifications applied
Details of DNase or RNAse treatment (E)
DNase treatment performed as described in RNeasy Micro Kit handbook (DNase reagents included).
Contamination assessment (DNA or RNA) (E)

qPCR control based on RT- reaction - minimal difference of 5 Cq values between RT- and RT+ samples achieved in all samples across all assays (except *B2m* assay which were excluded from final analysis). DNA contamination assessed only in bulk samples, in the case of single cells the control applied only randomly (in limited number of samples). DNA background in single cells could be considered negligible: i) expected only single copy of target sequence, ii) the majority of primers designed over introns.

The measured gene set contained some genes known to have high number of processed pseudogenes (e.g. *Gapdh, Actb, Ppia*). To control the false positivity, RT minus controls for all bulk samples and limited number of single cells (n =15) were included in the sample set. The bulk samples treated by DNase showed the low signal in RT minus controls, i.e. delta $Cq_{(\text{reverse transcribed - untranscribed samples})} > 5$, except the *B2m* assay, which were therefore excluded from further analysis. RT minus single cell controls were positive in the case of the *Actb*, *B2m*, *Gapdh*, and *Ppia* assays. Since the *Actb* and *Ppia* assays showed the rate of false positivity less than 5%, they were kept for further analysis, contrary to the highly positive *B2m* and *Gapdh* assays, which were excluded from further analysis.

Nucleic acid quantification (E)

RNA concentration in bulk samples quantified spectrophotometrically using NanoNodrop 1000 (ThermoScientific), in single cell not applied

Instrument and method (E)

NanoNodrop 1000 (ThermoScientific)

RNA integrity method/instrument (E)

not applied

RIN/RQI or Cq of 3' and 5' transcripts (E)

not applied

Inhibition testing (Cq dilutions, spike or other) (E)

spike assay

REVERSE TRANSCRIPTION

Complete reaction conditions (E)

- SuperScript® III Reverse Transcriptase used for RT-PCR
- to the lysed single cells in 5 µl NFW containing BSA added: 0.5 µl NFW (Life Technologies), 0.5 µl dNTP (10 mM, Invitrogen) and 0.5 ul primer mix - oligo-dT + random hexamers (50 µM, Eastport)
- mix incubated at 70 °C for 5 min, followed by 25 °C for 20 s; then placed on ice

- further added 0.25 µl NFW (Life Technologies), 2 µl 5X first-strand buffer (250 mM Tris-HCl (pH 8.3), 375 mM KCl, 15 mM MgCl2; provided with SSIII RT;), 0.5 µl DTT (100 mM; provided with SSIII RT), 0.5 µl RNase OUT (40 U/µl, Life Technologies) and 0.25 µl SSIII RT (200 U/µl, Life Technologies)
- mix incubated at 25 °C for 5 min, then at 50 °C for 60 min, followed by 55 °C for 15 min and finally terminated by heating at 75 °C for 15 min.
- 4 µl of RNA extracted from bulk samples were reverse transcribed in two 10-µl reactions into cDNA using the standard protocol of SuperScript III Reverse Transcriptase (Life Technologies)

Amount of RNA and reaction volume (E)

single cells - 5 ul RNA (concentration unknown), bulk samples - 4 µl RNA (5-10 ng/µl); both 10-µl reactions

Priming oligonucleotide (if using GSP) and concentration (E)

mix of random hexamers and oligodT primers (dT15) - both 0.5 µl of 50 µM

Reverse transcriptase and concentration (E)

SuperScriptIII™ reverse transcriptase - 50 U

Temperature and time (E)

1. step - 70 °C for 5 min, followed by 25 °C for 20 s; then placed on ice
2. step - 25 °C for 5 min, then at 50 °C for 60 min, followed by 55 °C for 15 min and finally terminated by heating at 75 °C for 15 min

Manufacturer of reagents and catalogue numbers (D)

SuperScript® III Reverse Transcriptase (18080044, Life Technologies)

Cqs with and without reverse transcription (D)

Cqs with reverse transcription < 40; Cqs without reverse transcription - undetected or difference higher difference than 5 Cqs between samples with and without reverse transcription

Storage conditions of cDNA (D)

In a freezer at -25 °C

qPCR TARGET INFORMATION

Gene symbol (E)

see Supplementary Table S1

Sequence accession number (E)

see Supplementary Table S1

Location of amplicons (D)

various; usually the middle part of mRNAs, or the part covering all splice variants

Amplicon length (E)

see Supplementary Table S1

In silico specificity screen (BLAST, etc) (E)

NCBI/ Primer-BLAST

Location of each primer by exon or intron (if applicable) (E)

see Supplementary Table S1

What splice variants are targeted? (E)

if possible primers designed to cover all splice variants; if not - primers designed to cover the highest number of splice variants as possible (in such cases usually tested more pairs of primers, covering different groups of similar splice variants, and finally chosen the one achieving the lowest Cq)

qPCR OLIGONUCLEOTIDES

Primer sequences (E)

see Supplementary Table S1

Location and identity of any modifications (E)

not applied

Manufacturer of oligonucleotides (D)

Invitrogen

Purification method (D)

desalted

qPCR PROTOCOL

Complete reaction conditions (E)

The qPCR protocol consisted from two separate steps: pre-amplification and qPCR. The purpose of pre-amplification is to increase the concentration of target molecules to be able to use the high throughput qPCR platform BioMark manufactured by Fluidigm (the pre-amplification step is mandatory because of minimal reaction volume 6.7 nl).

Pre-amplification was performed in two 40-µl reaction mixes, each containing 48 pairs of primers (for sequences and distribution into the two mixes, see Supplemental Table 1). Each pre-amplified reaction contained 20 µl of iQ Supermix (BioRad), 4 µl of a mix of 48 primers

(final concentration 25 nM each), 4 μl of cDNA (non-diluted for single cells; 2-times diluted for bulk samples), and water added to a final volume of 40 μl. The temperature profile was 95°C for 3 min followed by 18 cycles of amplification (95°C for 20 s, 57°C for 4 min and 72°C for 20 s on a Biorad CFX96). The two pools of pre-amplified cDNA were mixed, diluted (2.5-times for single cells; 5-times for bulk samples), snap frozen, and stored until analyzed in the BioMark platform. The pre-amplification protocol was verified on samples from three animals.

The qPCR was performed using The BioMark 96.96 Dynamic Array (Fludigm). The sample assay had a volume of 5 μl and contained 2 μl of diluted preamplified cDNA, 0.25 μl of DNA Binding Dye Sample Loading Reagent (Fluidigm), 2.5 μl SsoFast EvaGreen Supermix (Biorad), 0.01 μl ROX (Invitrogen), and 0.24 μl nuclease free water. The primer assay had a final volume of 5 μl and contained 2.5 μl Assay Loading Reagent (Fluidigm) and 2.5 μl of a mix of reverse and forward primers, corresponding to a final concentration of 4 μM. The cycling program was 3 min at 95°C for activation, followed by 35 cycles of denaturation at 95°C for 15 s, annealing at 60°C for 20 s, and elongation at 72°C for 20 s. After PCR, melting curves were collected between 60°C and 95°C with 0.5°C increments.

Reaction volume and amount of cDNA/DNA (E)

pre-amplification - 40-μl reactions; 4 μl cDNA (non-diluted for single cells; 2-times diluted for bulk samples)

qPCR - 6.7-nl reactions; 2 μl of diluted preamplified cDNA (2.5-times for single cells; 5-times for bulk samples) in the 5-μl sample assay (during chip-loading mixed with the primer assay 9:1)

Primer, (probe), Mg^{2+} and dNTP concentrations (E)

pre-amplification - 25 nM, iQ Supermix (BioRad)

qPCR - 400 nM; SsoFast EvaGreen Supermix (BioRad)

Polymerase identity and concentration (E)

a part of commercial mixes

Buffer/kit identity and manufacturer (E)

pre-amplification - iQ Supermix (BioRad)

qPCR - SsoFast EvaGreen Supermix (BioRad)

Additives (SYBR Green I, DMSO, etc.) (E)

not applied

Manufacturer of plates/tubes and catalog number (D)

pre-amplification - Multiplate™ 96-Well PCR Plates, high-profile, unskirted, clear, MLP9601 (BioRad)

qPCR - The BioMark 96.96 Dynamic Array (Fluidigm)

Complete thermocycling parameters (E)

pre-amplification - 95°C for 3 min followed by 18 cycles of amplification (95°C for 20 s, 57°C for 4 min and 72°C for 20 s)

qPCR - 3 min at 95°C for activation, followed by 35 cycles of denaturation at 95°C for 15 s, annealing at 60°C for 20 s, and elongation at 72°C for 20 s

Reaction setup (manual/robotic) (D)

manual

Manufacturer of qPCR instrument (E)

pre-amplification - CFX96 (Biorad)

qPCR - BioMark (Fludigm)

qPCR VALIDATION

Specificity (gel, sequence, melt, or digest) (E)

gel electrophoresis + melting curve analyses

For SYBR Green I, Cq of the NTC (E)

No signal in NTCs (tested 45 cycles)

Calibration curves with slope and y-intercept (E)

standard dilution series using specific PCR product over 6 orders of magnitude performed for each assay, from slopes calculated efficiencies (see Supplemental Table 1)

PCR efficiency calculated from slope (E)

see Supplementary Table S1

r^2 of calibration curve (E)

for all assays $r^2 < 0.99$

Linear dynamic range (E)

not determined; standard dilution series using specific PCR product over 6 orders of magnitude performed for each assay

Cq variation at limit of detection (E)

not determined

Evidence for limit of detection (E)

not determined

If multiplex, efficiency and LOD of each assay (E)

not applied

DATA ANALYSIS

qPCR analysis program (source, version) (E)

Bio-Rad CFX Manager 3.1; Fluidigm Real-Time PCR Analysis 4.1.2; GenEx Enterprise 6.0.1.612 (MultiD Analyses)

Method of Cq determination (E)

manual setting of threshold line in exponential phase of amplification curve (the same value for all assays)

Outlier identification and disposition (E)

not determined

Results of NTCs (E)

no signal in NTC

Justification of number and choice of reference genes (E)

reference genes (*Actb, Gapdh, Ppia, Hprt1, Ywhaz, Pgk1*) identified by NormFinder (Andersen et al. Cancer Res. 2004;64(15):5245-50); see Supplemental Text 2

Description of normalization method (E)

positive or negative shift of Cq values, according to the differences observed in reference genes, so the average of all Cq values did not change

Number and stage (RT or qPCR) of technical replicates (E)

bulk samples - 3 biological replicates, 1 RNA extraction replicate, 2 RT replicates; 1 pre-amplification replicates, 2 qPCR replicates

single cells samples - 32-81 biological replicates (see Table 1), following steps without technical replicates

Repeatability (intra-assay variation) (E)

not determined

Statistical methods for results significance (E)

Multivariate Analysis of Variance (MANOVA) using R software

Software (source, version) (E)

R version 3.1.3
